# Supplementary material for: Understanding X-ray absorption in liquid water using triple excitations in multilevel coupled cluster theory
Source: Nat Commun. 2024 Apr 26;15:3551. doi: 10.1038/s41467-024-47690-x (PMC11053016; doi:10.1038/s41467-024-47690-x)
Supplement: Supplementary file 1 — Supplementary Information [file 41467_2024_47690_MOESM1_ESM.pdf]

# Supplementary Information for "Understanding X-ray absorption in liquid water using triple excitations in multilevel coupled cluster theory"

Sarai Dery Folkestad[1†], Alexander C. Paul[1†], Regina Paul (née Matveeva)[1†], Sonia Coriani[2], Michael Odelius[3], Marcella Iannuzzi[4], Henrik Koch[1*]

[1]Department of Chemistry, Norwegian University of Science and Technology, NTNU, 7491 Trondheim, Norway.
[2]Department of Chemistry, Technical University of Denmark, DTU, 2800 Kongens Lyngby, Denmark.
[3]Department of Physics, Stockholm University, 10691 Stockholm, Sweden.
[4]Department of Chemistry, University of Zurich, 8057 Zürich, Switzerland.

*Corresponding author(s). E-mail(s): henrik.koch@ntnu.no;
†These authors contributed equally to this work.

**Keywords:** liquid water, X-ray spectroscopy, multilevel coupled cluster, coupled cluster, water clusters

## Source data

The source data can be accessed on Zenodo.

**Supplementary Table 1**: Contents of the source data folder available at Zenodo.

| Item | Contents |
|---|---|
| outputs_ccsd.tar.gz | eT output files for each geometry used containing CCSD-in-HF excitation energies and oscillator strengths for 45 excited states each. |
| outputs_mlcc3.tar.gz | eT output files for each geometry used containing MLCC3-in-HF excitation energies and oscillator strengths for 45 excited states each. |
| water_clusters_xyz.tar.gz | xyz-files containing the geometries of the water clusters used. The first 3 atoms are the central water molecule and the other water molecules are sorted by distance |
| density_analysis_mlcc3.tar.gz | eT output files for half the geometries containing MLCC3-in-HF excitation energies, oscillator strengths, and the charge transfer numbers for 45 excited states each. |
| source_data.zip | csv files containing the data required to reproduce the Figures in both main manuscript and Supplementary information. The files usually contain several columns listing the excitation energies and the corresponding intensities. |

# 1 Supplementary Figures

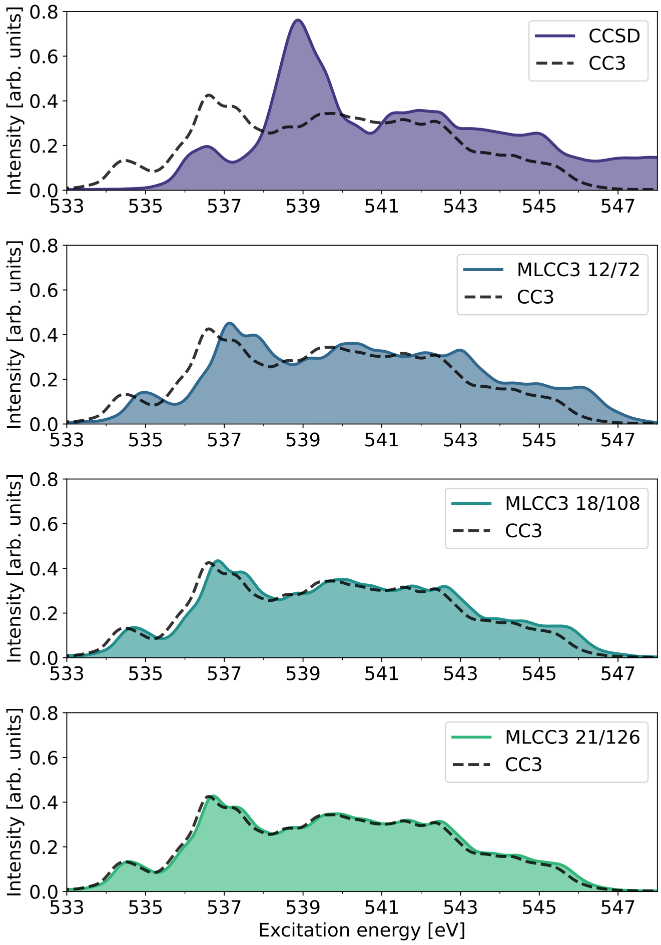


**Supplementary Figure 1**: **Convergence with CC3 space:** Averaged X-ray absorption spectra of 28 different water clusters at the MLCC3-in-HF level of theory showing the effect of including more CNTOs in the MLCC3 orbital space. The notation used in the legend indicates the number of occupied CNTOs before the slash and the number of virtual CNTOs after it. The MLCC3-in-HF excitations are broadened using Voigt profiles with 0.2 eV Lorentzian width and 0.2 eV Gaussian standard deviation (∼0.59 eV fwhm). The dashed line shows the spectrum obtained for a CC3-in-HF calculation, i.e. including all orbitals of the central 5 water molecules in the CC3 calculation.

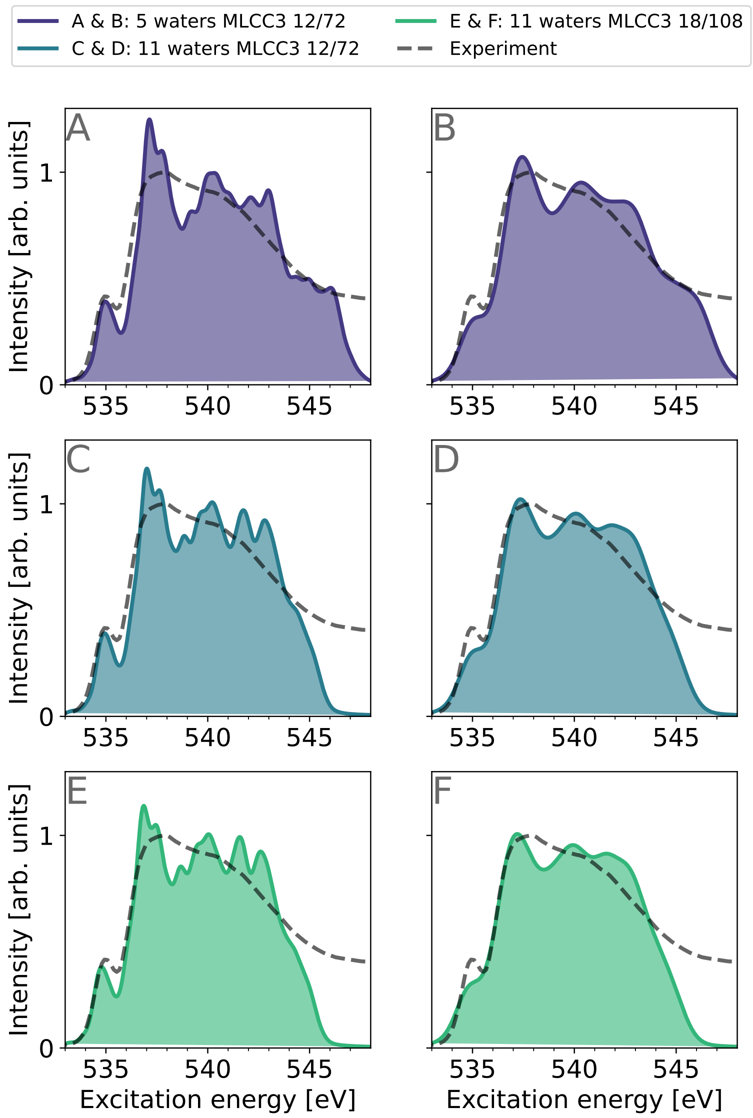


**Supplementary Figure 2**: **Enlarging both the CCSD and CC3 space:** X-ray absorption spectra averaged over 28 different water clusters at the MLCC3-in-HF level of theory showing the effect of including more waters in the coupled cluster space and increasing the number of orbitals in the MLCC3 space. For the first row (panel A, B) the CCSD space includes the central water and the four closest neighbouring water molecules and the MLCC3 space contains 12 occupied and 72 virtual CNTOs. For the second row (panel C, D) the 10 closest neighbours were included keeping the number of CNTOs. For the third row (panel E, F) the 10 closest neighbours were included and the number of CNTOs was increased to 18 occupied and 108 virtual CNTOs. In the left column the default broadening was used. In the right column the Gaussian standard deviation in the Voigt profiles was set to 0.5 eV increasing the overall full width at half maximum (FWHM) to $\sim$1.29 eV to further smooth the spectra.

## Difference densities

To visualize the importance of the environment for different parts of the spectrum, we plot the difference densities for selected states of a single snapshot, see Fig. 3. The first excited state is largely localized on the central water. Therefore, the difference density between the first excited state and other excited states shows the degree of delocalization of the higher excited state. Comparing the density differences between excited states 1 and 4 (left panel of Fig. 3) and excited states 1 and 34 (right panel of Fig. 3), we can see a gradual extension of the density from the central water to neighboring molecules. This supports the results of the charge transfer analysis that moving from the pre-edge to the post-edge, the character of the excitation becomes more diffuse.

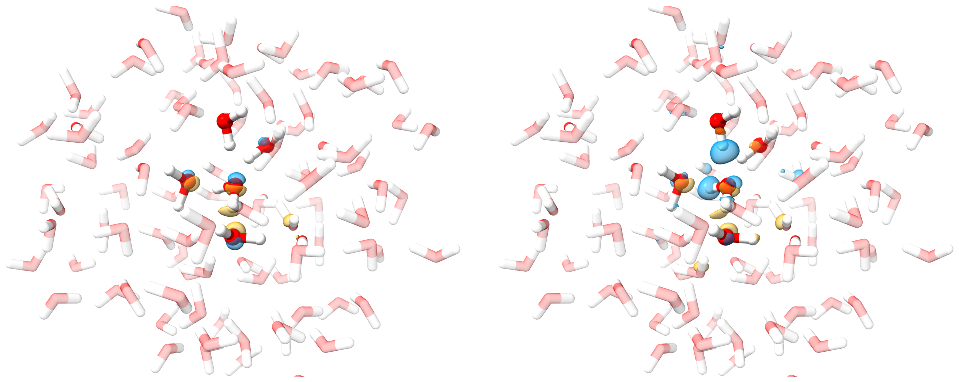

**Supplementary Figure 3**: **Difference densities for selected states:** Left: Difference of the MLCC3-in-HF excited state densities of excited states 4 and 1. Right: Difference of the MLCC3-in-HF excited state densities of excited states 34 and 1. The geometry of snapshot 68 step 01 was used and the iso-value was set to $4 \times 10^{-3}$ electrons bohr$^{-3}$. The yellow areas indicate a depletion of electron density in the transition from the first excited state (state 1) to the final state (either state 4 or state 34), and the blue areas indicate an addition of electron density.

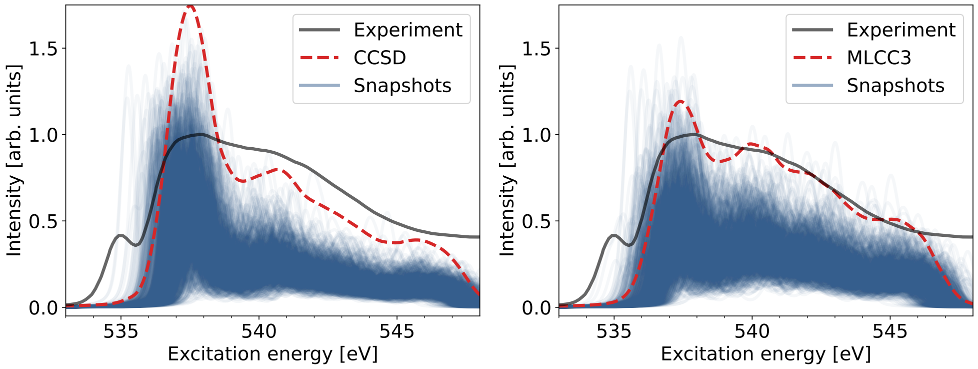


**Supplementary Figure 4**: **Spectra without first excitation:** X-ray absorption spectra of water clusters at the CCSD-in-HF (left) and MLCC3-in-HF (right) level of theory. The red, dashed lines show the spectra averaged over 896 individual snapshots (blue). The first excitation of each snapshot has been disregarded in this plot to identify the character of the pre-edge. The CCSD-in-HF spectrum was shifted by $-1.5\,\text{eV}$ to match the experiment. The experimental data was adapted from Ref. 1.

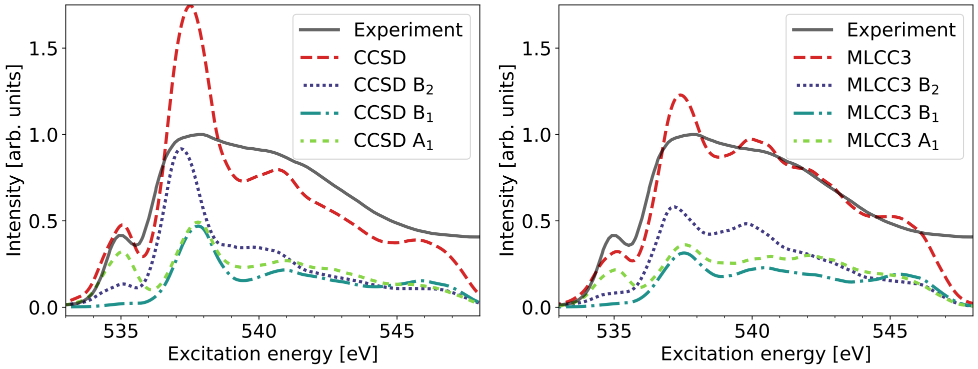


**Supplementary Figure 5**: **Symmetry decomposition of the spectrum:** X-ray absorption spectra of water clusters at the CCSD-in-HF (left) and MLCC3-in-HF (right) level of theory. The red lines show the complete spectra and the blue, turquoise, and green lines indicate the contributions of excitations that can be related to B2, B1 (out of plane) and A1 symmetry in a single water molecule. The CCSD-in-HF spectrum was shifted by $-1.5\,\text{eV}$ to match the experiment.

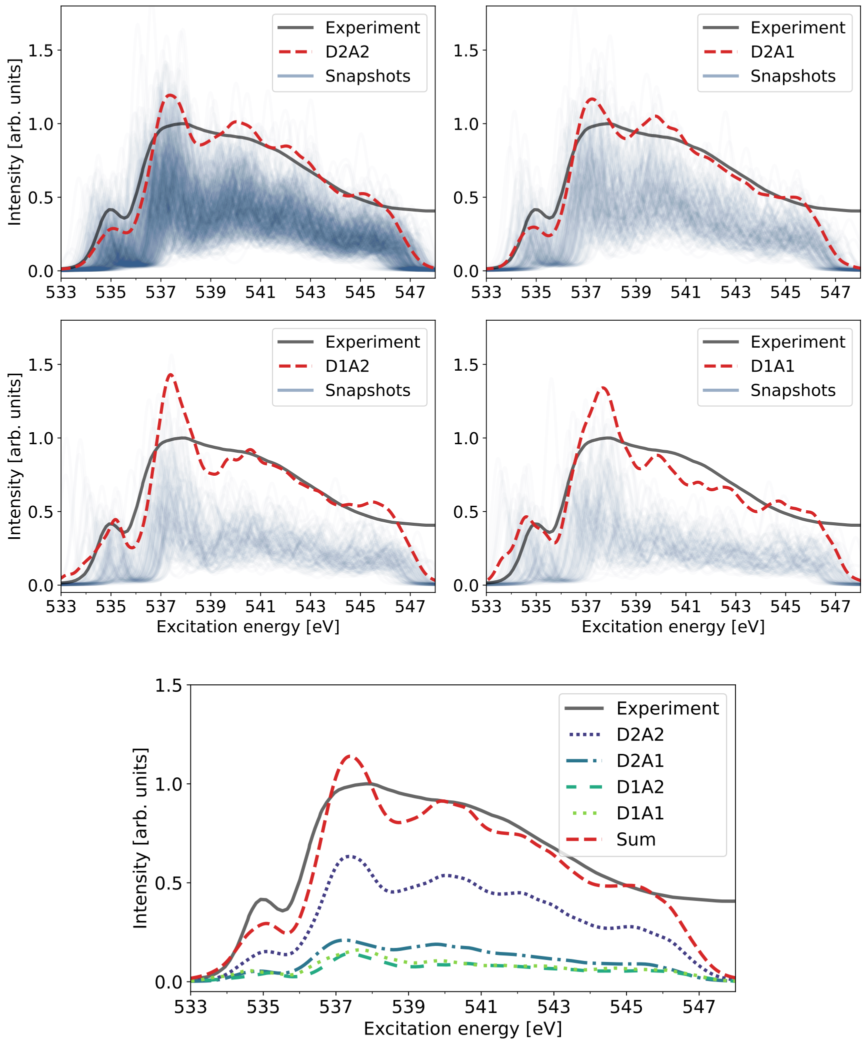


**Supplementary Figure 6**: **Hydrogen bond character decomposition of the spectrum:** X-ray absorption spectra of water clusters at the MLCC3-in-HF level of theory, divided by hydrogen bond character of the central water (D2A2, D2A1, D1A2, and D1A1). The hydrogen bond character is identified by the number of hydrogen bonds donated (D) and accepted (A) by the central water molecule. The calculated spectra were normalized to the experimental spectrum by matching the area under the curves in the range of $533 - 545\,\text{eV}$ and a Voigt broadening with Lorentzian FWHM of $0.2\,\text{eV}$ and Gaussian standard deviation of $0.2\,\text{eV}$ was used. The experimental data was adapted from Ref. 1. Top: The red, dashed lines show the spectra averaged over the individual snapshots of a given hydrogen bond character (blue). Bottom: Spectra scaled according to their importance D2A2: 0.53; D2A1: 0.18; D1A2: 0.12; D1A1: 0.10)

## Convergence with beads and snapshots

To evaluate the reliability of the geometries used in our calculations we investigate different choices for the sampling of the ab-initio path-integral molecular dynamics (PIMD) trajectory and compare with an ab initio molecular dynamics (AIMD) trajectory obtained using the same parameters. The spectra are computed with linear response time-dependent density functional theory (TDDFT) in the Tamm-Dancoff approximation, at the PBEh level of theory with the pcseg2 basis set using CP2K [2] with periodic boundary conditions [3]. The results are shown in Fig. 7.

First, we compare the spectra calculated for 70 geometries of the AIMD trajectory (denoted classical, panel E) with the spectrum obtained for 70 geometries from the PIMD trajectory denoted centroids (panel D). For the centroid geometries the hydrogen atoms are placed at the center of the 24-bead ring polymers. From the lower two panels of Fig. 7, we see that the spectra are virtually identical and only differ slightly in the intensity of the post-edge. However, both spectra exhibit significant differences compared to the spectrum obtained using the geometries of all beads for 70 snapshots (denoted All beads). By including quantum effects for the hydrogen atoms the spectra become smoother and the pre-edge intensity increases.

Considering all beads amounts to a large number of geometries. Therefore, we investigate a sampling of a single bead of the ring polymer instead of all beads. Calculating the spectrum for a single bead for 10 snapshots (panel C), already mostly reproduces the spectrum obtained when all beads are taken into account. When the number of snapshots is increased to 234 (panel A) or even only 70 (panel B), the difference becomes negligible. Therefore, we conclude that considering a number between 10 and 70 snapshots for a single bead should be sufficient to produce an accurate spectrum. In our calculations we considered 28 snapshots for which we calculate 896 individual spectra by sampling core excitations on each oxygen atom.

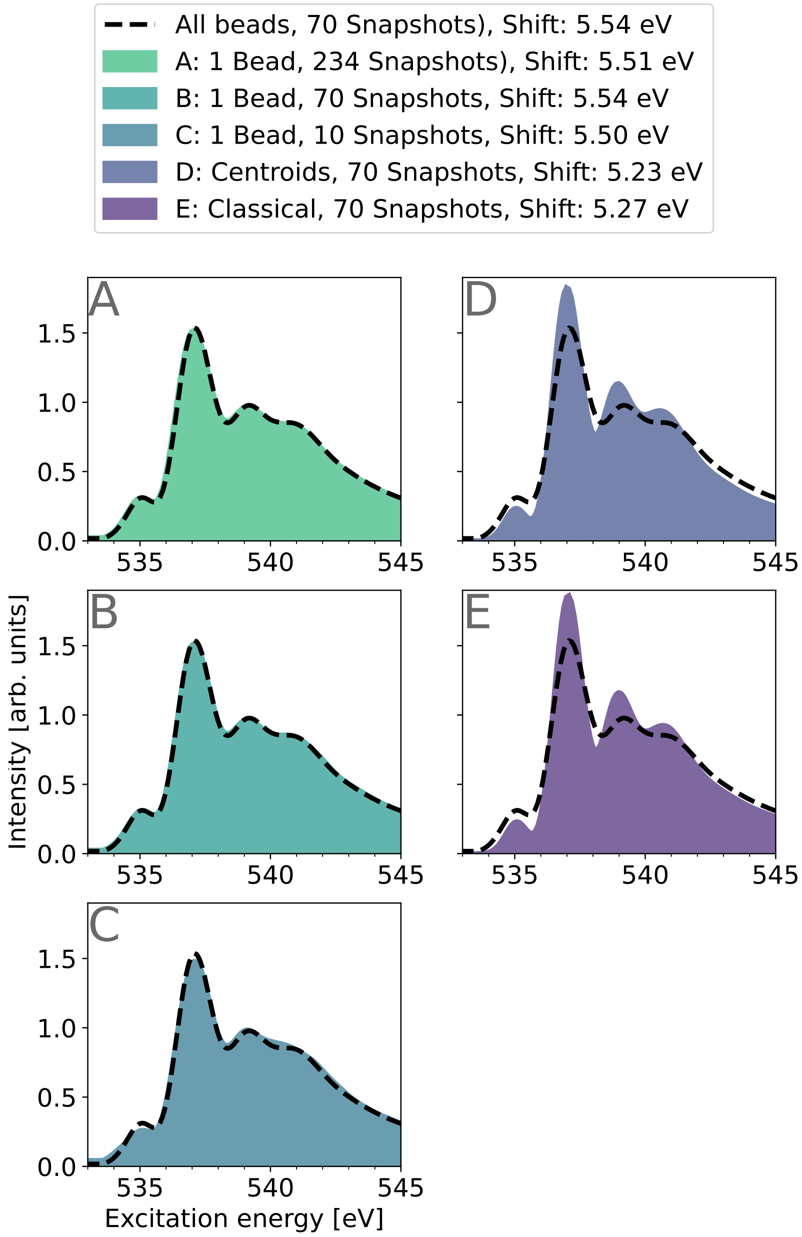


**Supplementary Figure 7**: **Convergence with beads and snapshots:** Comparison of the X-ray absorption spectra computed with linear response TDDFT using different selections of snapshots from the trajectory described in the main paper and another ab-initio molecular dynamics trajectory. The spectra were broadened using Gaussians of 0.8 eV width and normalized matching the area of the experimental spectrum in the range 534 eV to 544 eV. The spectra are averaged over the snapshots and the 32 oxygen atoms in the simulation box.

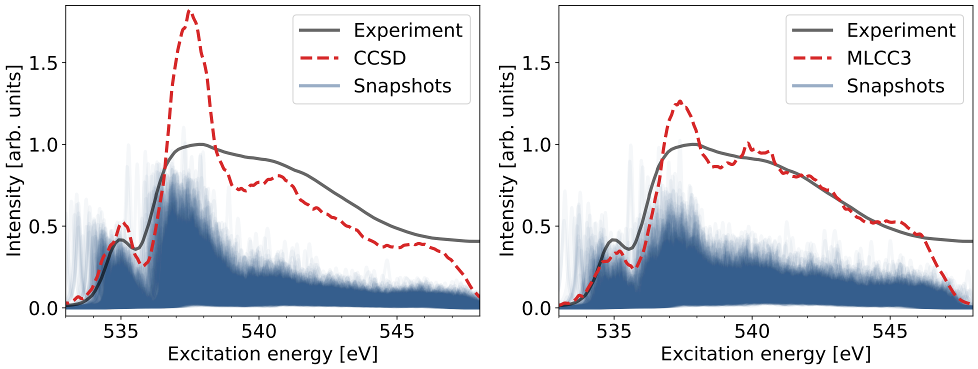


**Supplementary Figure 8**: **Lorentzian broadening:** X-ray absorption spectra of water clusters at the CCSD-in-HF (left) and MLCC3-in-HF (right) level of theory. The red lines show the spectra averaged over 896 individual snapshots (blue) The calculated spectra were normalized to the experimental spectrum by matching the area under the curves in the range of $533 - 545\,\text{eV}$ and a Lorentzian broadening with FWHM of $0.2\,\text{eV}$ was used. The CCSD-in-HF spectrum was shifted by $-1.5\,\text{eV}$ to match the experiment.

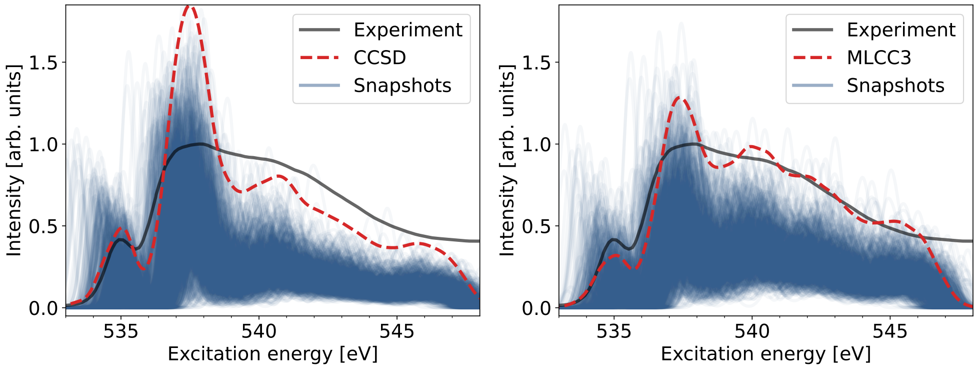


**Supplementary Figure 9**: **Gaussian broadening:** X-ray absorption spectra of water clusters at the CCSD-in-HF (left) and MLCC3-in-HF (right) level of theory. The red lines show the spectra averaged over 896 individual snapshots (blue) The calculated spectra were normalized to the experimental spectrum by matching the area under the curves in the range of $533 - 545\,\text{eV}$ and a Gaussian broadening with FWHM of $0.6\,\text{eV}$ was used. The CCSD-in-HF spectrum was shifted by $-1.5\,\text{eV}$ to match the experiment.

### Active orbital space

In Fig. 10 we show an example of the active space densities of the CCSD (left) and CC3 (right) active spaces (see Fig. 1). The active occupied density is plotted in blue and the virtual orbital density in yellow.

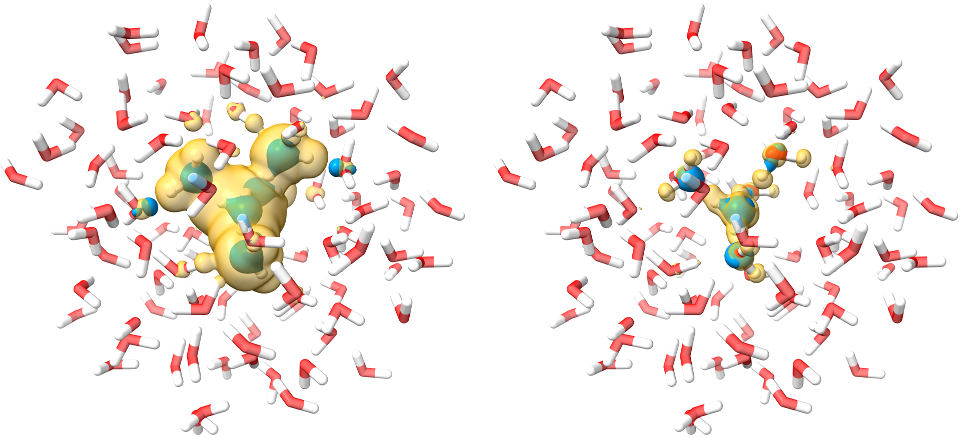

**Supplementary Figure 10**: **Visualization of the active spaces:** Left: Active occupied (blue) and virtual (yellow) CCSD-in-HF orbital densities, calculated from the active semi-localized MO. Right: Active occupied (blue) and virtual (yellow) MLCC3-in-HF orbital densities, calculated from the active CNTOs. An iso value of 0.1 electrons bohr$^{-3}$ is used in both plots.

## 2 Computational cost

The calculations were performed on the `bigmem` nodes of the cluster Saga of Sigma2, the provider of the national HPC infrastructure for computational science in Norway. These nodes consist of two Intel(R) Xeon(R) Gold 6138 CPU @ 2.00GHz with 20 threads each. The calculations were run using 20 threads for parallelization and a local scratch directory for improved `I/O`. Due to varying load on the nodes, the timings fluctuate significantly for different inputs which is reflected in the large standard deviation.

**Supplementary Table 2**: **Average time and memory usage:** Wall time and memory usage for the calculations run on Saga.

| | CCSD-in-HF | MLCC3-in-HF |
|---|---|---|
| Wall time [h] | $22.70 \pm 5.66$ | $23.22 \pm 5.64$ |
| Memory [GB] | $59.29 \pm 3.22$ | $62.00 \pm 0.67$ |

Note that the construction of the CNTOs in MLCC3-in-HF requires the CCSD-in-HF right excited state vectors. The time to construct the CCSD-in-HF excited state vectors is, however, not included in the MLCC3-in-HF timings because we restarted the MLCC3-in-HF calculations from the CCSD-in-HF results.

# 3 Comparison with GW-BSE

We compare our results with the results presented by Tang et al. in Ref. 4 using an iterative GW approximation in combination with the Bether-Salpeter equation (BSE). Both approaches offer state-of-the-art modeling of the XA spectrum of liquid water, but differ significantly in the theoretical framework employed. The GW-BSE approach belongs to the family of Green's function many-body perturbation theories, whereas CC-in-HF is a single-reference wave function-based method. In the study by Tang et al., periodic boundary conditions and a plane wave basis are used, while we consider water clusters with a standard $L_2$-basis. Additionally, Tang et al. approximately include contributions from high-lying valence excited states. These states are explicitly projected out in our CC-in-HF approach, where we employ the core-valence separation approximation.

To illustrate the differences between the experimental spectrum, our MLCC3-in-HF spectrum, and the GW-BSE spectrum by Tang et al., we added figure 11 showing the three spectra. Both theoretical spectra are in excellent agreement with the experimental one. Note that the GW-BSE spectrum has been shifted by 16 eV while the MLCC3-in-HF excitations are not shifted. Overall, the GW-BSE spectrum is smoother than the MLCC3-in-HF spectrum. However, the pre-edge is not as distinct as in the GW-BSE spectrum is not as distinct as in the experiment and starts to merge with the main edge. The post edge in the GW-BSE spectrum (around 542 eV) is overly intense, while the high energy region at approximately 545 eV has too little intensity.

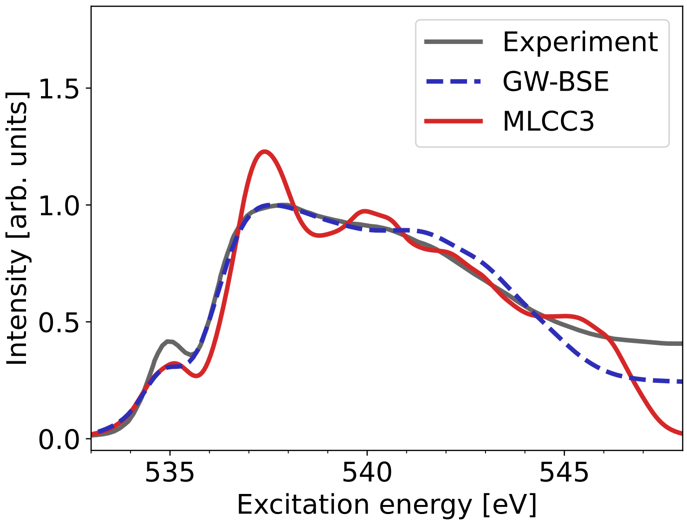


**Supplementary Figure 11**: **Comparison of MLCC3-in-HF and GW-BSE:** XAS spectra of water clusters at the MLCC3-in-HF level of theory (red) compared with the XAS spectrum from Fig. 1a of Ref. [4] calculated using $G_0W_0$-BSE@sc-$G^{static}W_0$ (blue), and the experimental spectrum (gray).

# References


[1] Sellberg, J.A., Kaya, S., Segtnan, V.H., Chen, C., Tyliszczak, T., Ogasawara, H., Nordlund, D., Pettersson, L.G.M., Nilsson, A.: Comparison of x-ray absorption spectra between water and ice: New ice data with low pre-edge absorption cross-section. J. Chem. Phys. **141**(3), 034507 (2014) https://doi.org/10.1063/1.4890035

[2] Kühne, T.D., Iannuzzi, M., Del Ben, M., Rybkin, V.V., Seewald, P., Stein, F., Laino, T., Khaliullin, R.Z., Schütt, O., Schiffmann, F., Golze, D., Wilhelm, J., Chulkov, S., Bani-Hashemian, M.H., Weber, V., Borštnik, U., Taillefumier, M., Jakobovits, A.S., Lazzaro, A., Pabst, H., Müller, T., Schade, R., Guidon, M., Andermatt, S., Holmberg, N., Schenter, G.K., Hehn, A., Bussy, A., Belleflamme, F., Tabacchi, G., Glöß, A., Lass, M., Bethune, I., Mundy, C.J., Plessl, C., Watkins, M., VandeVondele, J., Krack, M., Hutter, J.: CP2K: An electronic structure and molecular dynamics software package - Quickstep: Efficient and accurate electronic structure calculations. J. Chem. Phys. **152**(19) (2020) https://doi.org/10.1063/5.0007045 . 194103

[3] Bussy, A., Hutter, J.: Efficient and low-scaling linear-response time-dependent density functional theory implementation for core-level spectroscopy of large and periodic systems. Phys. Chem. Chem. Phys. **23**(8), 4736–4746 (2021) https://doi.org/10.1039/d0cp06164f

[4] Tang, F., Li, Z., Zhang, C., Louie, S.G., Car, R., Qiu, D.Y., Wu, X.: Many-body

effects in the X-ray absorption spectra of liquid water. Proc. Natl. Acad. Sci. **119**(20), 2201258119 (2022)
